# Simultaneous LV pressure-volume measurement in mice with MRI and ventricular catheterization

**DOI:** 10.1186/1532-429X-15-S1-W18

**Published:** 2013-01-30

**Authors:** A Wansapura, RS Dunn, J Wansapura

**Affiliations:** 1 Cincinnati Children's Hospital, Cincinnati, OH, USA

## Background

Stroke work normalized to end diastolic volume derived from pressure volume relationship is regarded as a gold standard for the measurement of the contractile status. Though conductance catheters are widely used to obtain pressure-volume relationships in the heart, they greatly underestimate end-diastolic and end-systolic volumes in mice. MRI on the other hand provides accurate measurement of left ventricular (LV) cavity volume but does not provide absolute measurement of ventricular pressure. The aim of this study was to demonstrate the feasibility of acquiring LV volume data while invasively measuring LV pressure in mice using an MR compatible fluid filled pressure transducer. Stroke work was calculated from the integrated area within the pressure-volume loop.

## Methods

Left ventricular pressure–volume relationships were obtained in three month old wild type C57BL/6 mice (n=5) and delta-sarcoglycan null (DSG) mice (n=3). Mice were anesthetized with an intraperitoneal injection of Ketamine and inactin and a tracheotomy was performed. To measure LV pressure in intact closed chest animals a fluid filled catheter connected to a low compliance COBE pressure transducer was inserted into the right carotid artery and advanced retrogradely across the aortic valve into the LV. Catheterized mice were then transferred to a 7 Tesla scanner for simultaneous pressure and volume measurements. Pressure–volume measurements were obtained at baseline resting state and during intraperitoneal infusion of beta adrenergic agonist Dobutamine (32ng/gBW/min).

## Results

The heart rate and the systolic pressure increased and the stroke volume decreased during stress. The approximate total scan time for volume data were 3.5 and 2 minutes during rest and stress respectively. The Stroke work of DSG decreased from 2.8±0.56 mmHg mL at rest to 1.1±0.49 mmHg mL at stress but did not change in WT significantly between at rest and at stress. The fluid filled pressure catheter while visible in the LV cavity did not introduce any appreciable artifacts.

## Conclusions

This study demonstrates for the first time that invasive pressure and MRI derived volume measurements can be made simultaneously in mice. Pressure-volume data obtained using this technique could provide important information about the contractile status of the mouse heart independent of the loading condition. The decline in stroke work in DSG under adrenergic stimulation may points to heart failure in DSG mice even at 12 weeks of age.

## Funding

This work was supported by the NIH Heart, Lung and Blood Institute grant K25HL102244.

**Figure 1:**
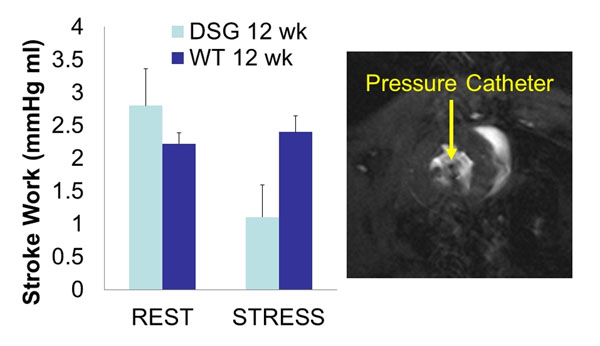
Stroke work of DSG is decreased with adrenergic simulation.

